# Combined ultrasound–CT approach to monitor acute exacerbation of interstitial lung disease

**DOI:** 10.1186/s13089-020-00174-7

**Published:** 2020-05-15

**Authors:** Silvia Mongodi, Andrea Colombo, Anita Orlando, Lorenzo Cavagna, Bélaid Bouhemad, Giorgio Antonio Iotti, Francesco Mojoli

**Affiliations:** 1 Anaesthesia and Intensive Care, San Matteo Hospital, Viale Golgi 19, Pavia, Italy; 2 Department of Clinical-Surgical, Diagnostic and Paediatric Sciences, Unit of Anaesthesia and Intensive Care, University of Pavia, Pavia, Italy; 3 Division of Rheumatology, University of Pavia, San Matteo Hospital, Pavia, Italy; 4 Dijon and Université Bourgogne Franche-Comté LNC UMR866, F-21000 Dijon, BP 77908, Dijon Cedex, 21709 France; 5 Department of Anesthesiology and Intensive Care, C.H.U. Dijon, Dijon Cedex, France

**Keywords:** Interstitial lung disease, Lung ultrasound, High-resolution CT, ECMO, Lung monitoring

## Abstract

**Background:**

Lung ultrasound is a bedside non-irradiating tool for assessment and monitoring of lung diseases. A lung ultrasound score based on visualized artefacts allows reliable quantification of lung aeration, and is useful to monitor mechanical ventilation setting, fluid resuscitation and antibiotic response in critical care. In the context of interstitial lung diseases associated to connective tissue disorders, lung ultrasound has been integrated to computed tomography for diagnosis and follow-up monitoring of chronic lung disease progression.

**Case presentation:**

This case describes a severe acute exacerbation of interstitial lung disease associated to dermatomyositis–polymyositis requiring prolonged extra-corporeal life support. Lung ultrasound score was performed daily and allowed monitoring and guiding both the need of advanced imaging as computed tomography and immunosuppressive therapy.

**Conclusions:**

This case suggests lung ultrasound may be a useful monitoring tool for the response to immunosuppressive therapy in acute severe rheumatic interstitial lung disease, where chest X-ray is poorly informative, and transportation is at high risk.

## Background

Lung ultrasound is increasingly used to assess and monitor pulmonary diseases in critically ill patients [1]. Although computed tomography scan (CT) represents the gold standard for the majority of lung pathologies, it is burdened with several limitations as exposure to ionizing radiations and need of transportation to radiology department. Lung ultrasound helps in the differential diagnosis of acute respiratory failure [2], reducing ionizing radiation and relative medical costs [3]. In particular, ultrasound-aided diagnostic criteria for ARDS have been proposed [4]. Moreover, a lung ultrasound score can be computed to quantify lung loss of aeration, with strong association with quantitative CT assessment [5]. In ARDS patients, lung ultrasound score may guide positive end-expiratory pressure titration [6] and fluid resuscitation [7] and can be used for daily monitoring of lung recovery [8]. In critically ill patients with ventilator-associated pneumonia, it has been applied to monitor the response to antibiotics [9].

In the context of interstitial lung disease (ILD) associated to connective tissue diseases, lung ultrasound has been proposed as an integration to CT for diagnosis [10] and follow-up monitoring [11]; in this chronic setting, different quantitative approaches based on the total number of visualized B-lines in all the intercostal spaces have been applied to monitor ILD progression [12] and evaluate timing of radiological follow-up [13]. However, how to use lung ultrasound to monitor the response to treatment in acute respiratory failure associated to rheumatic diseases is still unclear. We describe a prolonged ultrasound monitoring in acute exacerbation of ILD associated to connective tissue disease.

### Case presentation

**Fig. 1:**
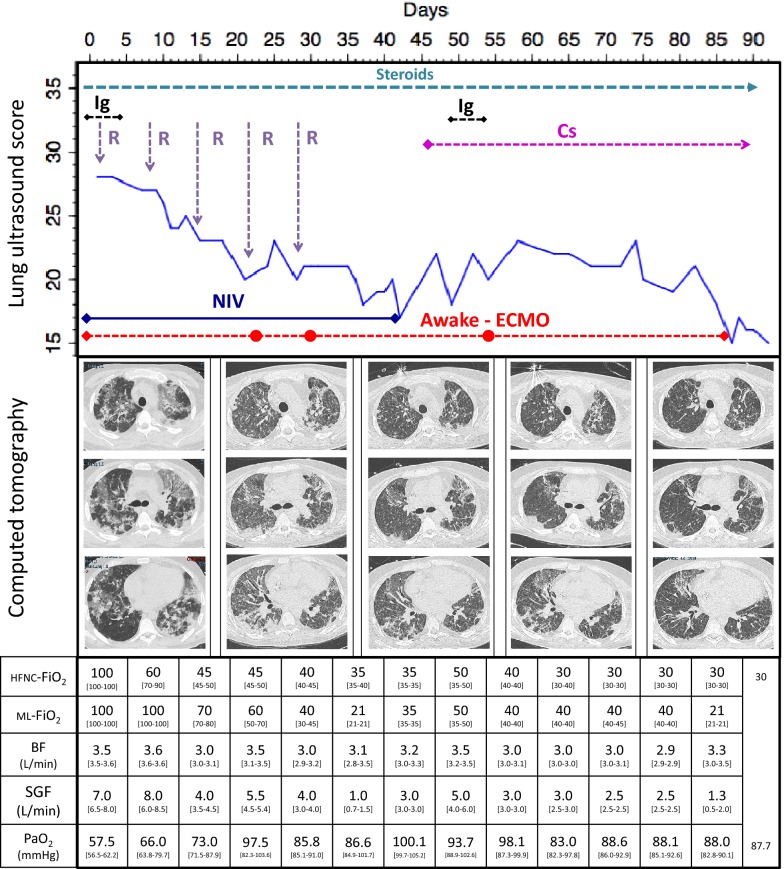
Lung ultrasound score trend, computed tomography and clinical parameters during 93 days of extra-corporeal life support for acute exacerbation of interstitial lung disease in dermatomyositis–polymyositis (*R* rituximab, *Ig* immunoglobuline, *Cs* cyclosporine, *NIV* non-invasive ventilation, *ECMO* extra-corporeal membrane oxygenation—red dot: membrane lung replacement, *HFNC-FiO*_*2*_ fraction of inspired oxygen delivered by high flow nasal cannula, *ML-FiO*_*2*_ fraction of inspired oxygen delivered by the membrane lung, *SGF* sweep gas flow, *PaO*_*2*_ patient’s partial arterial oxygen pressure)

A 28-year-old man affected by dermatomyositis–polymyositis was admitted to our general intensive care unit for hypoxemic respiratory failure. He had been diagnosed with dermatomyositis–polymyositis 1 year before after muscular and cutaneous symptoms, had been treated with cyclophosphamide and was under chronic corticosteroid therapy. In intensive care unit, he was first supported with high-flow nasal cannula (HFNC) oxygen therapy. Infectious aetiology was ruled out by negative microbiological specimens (bronchoalveolar lavage for virus, bacteria and parasites, haemocultures, uroculture, serology for *Chlamydia*, *Legionella* and *Mycoplasma Pneumoniae*, urinary antigens for *Legionella* and *Streptococcus Pneumoniae*). A transthoracic echocardiogram was performed but no alterations of the left ventricular systolic and diastolic function were found (ejection fraction > 55%). Patient was also negative for acute or chronic valvular diseases. Autoimmune analyses showed anti-nuclear antibody positivity (speckled pattern 1:160) and weak reactivity of anti-melanoma differentiation-associated protein 5 antibodies. Immunosuppressive therapy with high-dose intravenous immunoglobulin (2 g/kg/day for 5 days, then 0.4 g/kg/day) and methylprednisolone (1 g daily for 3 days, then 40 mg twice daily) was then started. Nevertheless, he rapidly deteriorated and required intubation for refractory hypoxemia [partial arterial oxygen pressure (PaO_2_) 63.4 mmHg with fraction of inspired oxygen (FiO_2_) 0.8 by HFNC, pH 7.45, PaCO_2_ 45.3 mmHg]. A second-line immunosuppressive treatment was introduced, with rituximab (375 mg/m^2^ once a week for five cycles) and plasma exchange. Veno-venous femoro-jugular extracorporeal membrane oxygenation (ECMO) was also placed percutaneously to extubate the patient and reduce the risk of overinfection whilst immunosuppressed. Once the ECMO started and the patient extubated, non-invasive respiratory support was provided by HFNC associated to 2–3 daily cycles of mask non-invasive ventilation. Initial CT showed multiple parenchymal consolidations and diffuse ground-glass pattern affecting all lobes, bilateral architectural distortion and nodular-pattern blurred opacities with partial sparing of inferior right lobe. Lung ultrasound score was performed daily to monitor ILD response to immunosuppressive therapy, being chest X-ray poorly informative in an almost completely white lung and being transportation to CT at particularly high risk for both immunosuppression and extra-corporeal support. 12 standard thoracic regions were examined in supine position with thorax elevation around 45°, using in transversal scan a 10-MHz linear probe or a 2.5-MHz phased-array probe when a tissue-like pattern was visualized. Four steps of progressive loss of aeration were distinguished; each region was scored from 0 (normal aeration) to 3 (complete aeration loss), according to the visualized pattern, as previously described [5–9, 14]. The sum of each region’s score gives the global lung ultrasound score [1, 5–8], therefore ranging from 0 to 36. Lung ultrasound score, HFNC FiO_2_, ECMO settings and patient’s PaO_2_ are shown in Fig. 1. Initial lung ultrasound score was 28; a progressive reduction of the score from 28 to 17 was observed in the first 5 weeks, confirming the positive response to immunosuppressive therapy, as also supported by the CT performed after 15 days. Gas exchanges also improved with reduced ECMO and inspired FiO_2_. However, a complete lung recovery was not observed; the lung ultrasound score ranged between 17 and 22 for days and the patient failed two tests of ECMO discontinuation. A third CT was then performed, where evolution to fibrosis was described. Once overinfection was ruled out again, a third-line immunosuppressive therapy with a second cycle of intravenous immunoglobulin and intravenous cyclosporine (100 mg twice daily) was introduced. Non-invasive respiratory approach was also changed, pursuing with HFNC only. After an initial worsening, a second slower reduction in lung ultrasound score was observed, corresponding to better lung aeration at CT. The patient was finally weaned from ECMO after 93 days with a lung ultrasound score of 15 and good gas exchanges in HFNC (PaO_2_ 96.1 mmHg with FiO_2_ 0.3, pH 7.38, PaCO_2_ 51.2 mmHg).

## Conclusions

Lung ultrasound score and CT were here combined to assess and monitor lung aeration of acute exacerbation of dermatomyositis–polymyositis. CT provides the best assessment of ILD and optimal quantification of lung aeration loss; however, for its costs, irradiation and risk related to transportation of critical patients it cannot be considered a monitoring tool. Lung ultrasound score is a reliable alternative for quantification of lung aeration [5]; it can reduce the radiation exposure in critically ill patients, can be performed bedside and requires few minutes for a complete examination in expert hands. In this case of prolonged extra-corporeal support, the need of advanced imaging was triggered by lung ultrasound findings; this led to a very limited number of CTs. Whilst the absolute value of lung ultrasound score does not provide clear information, it can be useful if compared to initial CT and to monitor lung aeration variations in response to treatments finalized at pulmonary aeration improvement, as mechanical ventilation setting [6], antibiotics [9] or, in case of rheumatic diseases, immunosuppressive therapy. Whilst more and more used for many common respiratory diseases in critical care [1], lung ultrasound in acute ILD associated to connective tissue diseases has been less investigated. In particular, the interest of lung ultrasound score for aeration monitoring in response to immunosuppressive therapy has not been explored yet in this context. This case suggests lung ultrasound may integrate the classical imaging and clinical management of acute exacerbations of ILD, providing useful daily information on lung aeration and on its response to immunosuppressive treatment, whilst integrating and eventually guiding the need of advanced imaging as CT.

## Data Availability

The datasets used and/or analysed during the current study are available from the corresponding author on reasonable request.

## Abbreviations

CT: Computed tomography
ECMO: Extra-corporeal membrane oxygenation
FiO_2_: Fraction of inspired oxygen
HFNC: High-flow nasal cannula
ICU: Intensive care unit
ILD: Interstitial lung disease
LUS: Lung ultrasound
PaO_2_: Partial arterial oxygen pressure
